# Chorioamnionitis: Association of Nonreassuring Fetal Heart-rate Patterns and Interval From Diagnosis to Delivery on Neonatal Outcome

**DOI:** 10.1155/S106474499400058X

**Published:** 1994

**Authors:** Paul J. Wendel, Susan M. Cox, Scott W. Roberts, Jody Dax, Larry C. Gilstrap

**Affiliations:** 1 Department of Obstetrics and Gynecology University of Texas Southwestern Medical Center Dallas TX USA; 2 Department of Obstetrics and Gynecology University of Arkansas for Health Sciences 4301 W. Markham Slot 518 Little Rock AR 72205 USA

## Abstract

*Objective:* The purpose of this study was to determine whether selected fetal heart-rate (FHR) patterns and the interval from diagnosis to delivery in pregnancies complicated by chorioamnionitis could predict neonatal outcome.

*Methods:* During a 6-month period, 217 consecutive patients with acute chorioamnionitis were prospectively identified in labor. Following delivery, the fetal monitor strips and hospital courses were reviewed for both the mother and neonate. Multiple logistic regression was used to analyze the presence of a nonreassuring FHR pattern and the effect on neonatal outcome. Fisher exact tests were used to analyze the time intervals from the diagnosis of chorioamnionitis to delivery and their significance on neonatal outcome parameters.

*Results:* The overall incidence of chorioamnionitis in our population was 2.3%. None of the independent variables analyzed following the diagnosis of chorioamnionitis until delivery were significantly associated with an umbilical artery (Ua) pH < 7.20. There were no differences in cord pH, Apgar scores, sepsis, admission to special-care nursery, and oxygen requirements in neonates based on the duration of time from the diagnosis of chorioamnionitis to delivery in our study. None of the newborns had pathologic fetal acidemia (Ua pH < 7.00). None of the FHR patterns we identified after the diagnosis of acute chorioamnionitis were significantly associated with neonates with a Ua pH < 7.20.

*Conclusions:* An interval from diagnosis to delivery of up to 12 h plays little if any role in neonatal outcome.


Infectious Diseases in Obstetrics and Gynecology 2:162–166 (1994)
© 1994 Wiley-Liss, Inc.

### Chorioamnionitis: Association of Nonreassuring Fetal Heart-Rate Patterns and Interval From Diagnosis to Delivery on Neonatal Outcome

**Paul J. Wendel, Susan M. Cox, Scott W. Roberts, Jody Dax, and Larry C. Gilstrap**

*Department of Obstetrics and Gynecology, University of Texas Southwestern Medical Center, Dallas, TX*

### Abstract

***Objective:*** The purpose of this study was to determine whether selected fetal heart-rate (FHR) patterns and the interval from diagnosis to delivery in pregnancies complicated by chorioamnionitis could predict neonatal outcome.

***Methods:*** During a 6-month period, 217 consecutive patients with acute chorioamnionitis were prospectively identified in labor. Following delivery, the fetal monitor strips and hospital courses were reviewed for both the mother and neonate. Multiple logistic regression was used to analyze the presence of a nonreassuring FHR pattern and the effect on neonatal outcome. Fisher exact tests were used to analyze the time intervals from the diagnosis of chorioamnionitis to delivery and their significance on neonatal outcome parameters.

***Results:*** The overall incidence of chorioamnionitis in our population was 2.3%. None of the independent variables analyzed following the diagnosis of chorioamnionitis until delivery were significantly associated with an umbilical artery (Ua) pH < 7.20. There were no differences in cord pH, Apgar scores, sepsis, admission to special-care nursery, and oxygen requirements in neonates based on the duration of time from the diagnosis of chorioamnionitis to delivery in our study. None of the newborns had pathologic fetal acidemia (Ua pH < 7.00). None of the FHR patterns we identified after the diagnosis of acute chorioamnionitis were significantly associated with neonates with a Ua pH < 7.20.

***Conclusions:*** An interval from diagnosis to delivery of up to 12 h plays little if any role in neonatal outcome. © 1994 Wiley-Liss, Inc.

Key words

Intraamnionic infection, neonatal acidemia, pregnancy

Acute chorioamnionitis is an infection of the amniotic fluid, placenta, placental membranes, uterus, and uterine contents. Other terms have been employed to describe this clinical scenario which include intraamniotic infection (IAI), amnionitis, and intrapartum infection. Acute chorioamnionitis is a relatively common complication of pregnancy, occurring in 0.5–2% of all deliveries.[1,2] Recently, even higher frequencies of such infections have been reported involving as many as 4–10.5% of pregnancies.[3,4] Maternal outcome is generally good; however, significant fetal and neonatal morbidity and mortality may be associated with chorioamnionitis.

Nonreassuring fetal heart-rate (FHR) patterns such as tachycardia and the absence of FHR variability are relatively common in the presence of acute chorioamnionitis.[1,5,6] Although these patterns

Address correspondence/reprint requests to Dr. Paul J. Wendel, Department of Obstetrics and Gynecology, University of Arkansas for Health Sciences, 4301 W. Markham, Slot 518, Little Rock, AR 72205.

Received July 7, 1994
Accepted September 28, 1994

have been utilized as markers of "fetal distress" or "fetal jeopardy" in women without chorioamnionitis, their significance as predictors of neonatal outcome in the presence of IAI is unclear. Moreover, there are little to no data regarding the significance of an absence of FHR accelerations in pregnancies with acute chorioamnionitis.

The purpose of this investigation was to determine if selected FHR patterns, i.e., loss of variability, degree of fetal tachycardia, or absence of FHR accelerations, could predict neonatal outcome in pregnancies complicated by chorioamnionitis. Additionally, we sought to determine whether there was an appropriate interval from the diagnosis of infection to delivery in which good neonatal outcome could be expected.

### SUBJECTS AND METHODS

Patients at Parkland Memorial Hospital (a city-county facility serving a predominantly indigent population) with the clinical diagnosis of chorioamnionitis were prospectively identified in labor over a 6-month period, October 1992 through March 1993. The diagnosis of chorioamnionitis was based primarily on the presence of fever defined as a temperature elevation of ≥38°C without an otherwise identifiable source of infection. Other criteria to support the diagnosis included fetal tachycardia, maternal tachycardia, and uterine tenderness. Once the diagnosis of chorioamnionitis was made, the patient was started on parenteral antibiotics and antipyretics as necessary for persistent fever of ≥38°C. All patients received ampicillin and gentamicin unless there was a history of penicillin allergy, in which case cefoxitin was utilized. All women were monitored by both an intrauterine pressure catheter and a fetal scalp electrode. The diagnosis of endometritis was made when fever and uterine tenderness persisted for ≥48 h following delivery.

Following delivery, the FHR tracings and hospital courses were reviewed for both the mother and neonate. Both the time of the diagnosis of chorioamnionitis and administration of intravenous (IV) antibiotics were noted. The review of the fetal monitor tracings was accomplished by 2 independent examiners. Each monitor strip was appropriately identified and matched with the appropriate times of diagnosis and treatment for chorioamnionitis. The monitor strips were then analyzed until delivery. Each 30-min interval was evaluated for the presence of long-term variability, degree of fetal tachycardia, number of FHR accelerations, and number and degree of decelerations. Examiners were blinded to fetal outcome, i.e., Apgars and umbilical blood gas results as well as neonatal hospital course.

FHR accelerations were identified if a 15-beat elevation over the established baseline lasted at least 15 s.[7] A long-term variability was assessed if beat-to-beat changes of 5 beats/min (bpm) occurred in >2 cycles/min.[7] The baseline FHRs were assessed as follows: normal (120–159 bpm), mild tachycardia (160–179 bpm), and severe tachycardia (≥180 bpm). FHR decelerations were classified as variable or late in nature. Only moderate and severe variables and late decelerations were analyzed for their significance on neonatal outcome. Moderate variable decelerations were those in which the heart rate fell below 70 bpm for 30–60 s or those between 70 and 80 bpm for >60 s.[7] Severe variable decelerations were defined as a fall in the FHR of <70 bpm for >60 s.[7] A late deceleration was a fall in the FHR beginning at or after the peak of the uterine contraction.[7] As previously defined, an umbilical artery (Ua) pH < 7.20 was considered low.[8–11] In the 197 mothers with chorioamnionitis, we sought to determine whether the time elapsed from diagnosis to delivery played a role in neonatal outcome. The interval categories from diagnosis to delivery were arbitrarily assigned to 0–2, 2–4, 4–6, 6–12, and 12–18 h.

Multiple logistic regression analysis was used to control for confounding variables. The independent variables included mild and moderate tachycardia, loss of FHR variability, lack of FHR accelerations, absence of severe and late decelerations, and birth weight <2,500 g. Exposure and outcome measures were compared with Fisher exact tests.

### RESULTS

During the 6 months noted previously, 217 mothers were identified with chorioamnionitis and followed. Of these, the majority (130 or 66%) were Hispanic, 42 (21%) were black, and 16 (8%) were white. Moreover, 155 (79%) were nulliparas and the majority (112 or 57%) were between ages 20 and 29 years. Twenty patients were excluded from analysis because the diagnosis of acute chorioamnionitis was unclear or antibiotics were not admin-

**TABLE 1. Diagnostic criteria for acute chorioamnionitis**

| | No. (%) |
|---|---|
| Fever[a] | 197 (100) |
| Fetal tachycardia | 143 (73) |
| Maternal tachycardia | 56 (28) |
| Maternal leukocytosis | 13 (7) |
| Foul-smelling amniotic fluid | 12 (6) |
| Uterine tenderness | 11 (5.5) |

[a]The major inclusion criterion.

istered until after delivery. In those in whom the diagnosis was unclear, either a temperature of ≥38°C had not been documented or another source of fever could not be excluded. Thus, 197 women with acute chorioamnionitis were included in this analysis. There were 9,268 deliveries during this period, resulting in an incidence of 2.3% for chorioamnionitis in our population. The antibiotics utilized for treatment in these 197 women with acute chorioamnionitis included ampicillin plus genatmicin (186 or 94%), cefoxitin plus gentamicin (6 or 3%), cefoxitin only (3 or 1.5%), and ampicillin plus gentamicin plus clindamycin (1 or 2%).

The diagnostic criteria for acute chorioamnionitis are summarized in Table 1. Since fever was the major inclusion criterion, all patients had a temperature of ≥38°C. Moreover, 73% of the fetuses manifested tachycardia. The FHR patterns of the 197 fetuses of women with acute chorioamionitis are summarized in Table 2. Mild FHR tachycardia was the most common FHR pattern (56%). An absence of FHR accelerations was encountered in 55 (28%) of the fetuses. Only 8 (4%) of the newborns had late decelerations during monitoring.

The results of Ua pH determinations are also summarized in Table 2. Almost three-fourths (72%) had a Ua pH ≥ 7.20. Of the 55 (28%) newborns with a pH < 7.20, 60% were in the 7.15–7.19 group.

Independent variables were analyzed according to a multiple logistic regression model.[12] The duration of exposure for this statistical model was based upon the time from diagnosis to delivery. Independent variables were assigned a relative risk with confidence intervals set at 95%. The independent variables analyzed for their association with neonatal acidemia included a birth weight of

**TABLE 2. FHR patterns and Ua pH determinations in 197 fetuses of women with acute chorioamnionitis**

| | No. (%) |
|---|---|
| FHR pattern | |
| Mild tachycardia (160–179 bpm) | 110 (56) |
| Absence of acceleration | 55 (28) |
| Normal baseline | 54 (27) |
| Severe tachycardia (≥180 bpm) | 33 (17) |
| Absence of variability | 9 (5) |
| Late deceleration | 8 (4) |
| Severe variability | 7 (4) |
| Ua pH | |
| ≥7.20 | 142 (72) |
| <7.20 | 55 (28) |
| 7.15–7.19 | 33 (60) |
| 7.10–7.14 | 9 (16) |
| <7.10 | 13 (24) |

**TABLE 3. Multiple logistic regression of variables with a Ua pH <7.20**

| | Relative risk | *P* | Confidence interval (95%) |
|---|---|---|---|
| Birth weight (<2,500 g) | 1.0 | 0.95 | (0.3–3.3) |
| Loss of variability | 3.2 | 0.22 | (0.5–20.6) |
| Absence of severe/late decelerations | 0.4 | 0.09 | (0.2–1.1) |
| No accelerations | 1.0 | 0.98 | (0.4–2.6) |
| Mild tachycardia (160–180 bpm) | 0.8 | 0.57 | (0.4–1.7) |
| Severe tachycardia (≥181 bpm) | 1.2 | 0.75 | (0.5–3.0) |

<2,500 g, a loss of FHR variability, an absence of severe and late decelerations, no FHR accelerations, and mild and severe fetal tachycardia. The results are summarized in Table 3. None of the variables we analyzed from the time of diagnosis to delivery were significantly associated with neonatal acidemia defined by a pH < 7.20.[8–11]

The results of neonatal outcome parameters and breakdown according to neonatal outcome are summarized in Table 4. There were no statistical differences in cord pH, sepsis, Apgar scores, admission to special-care nursery, and oxygen requirements in neonates based on the duration of time from the diagnosis of chorioamnionitis to delivery in our study.

Seventeen (9%) women had endometritis. There was no relation between this diagnosis and the duration of acute chorioamnionitis.

**TABLE 4. Neonatal outcome parameters according to the interval from the diagnosis of chorioamnionitis to delivery[a]**

| | Interval (h) | | | | | |
|---|---|---|---|---|---|---|
| | 0–2 | 2–4 | 4–6 | 6–12 | 12–18 | P |
| Number | 80 | 57 | 39 | 18 | 3 | — |
| Apgars at 5 min | | | | | | |
| ≤6 | 2 (3) | 3 (5) | 1 (3) | 1 (6) | 0 (0) | 0.72 |
| ≤3 | 0 (0) | 1 (2) | 0 (0) | 1 (6) | 0 (0) | 0.17 |
| Cord gas | | | | | | |
| pH < 7.20 | 25 (31) | 19 (33) | 17 (44) | 6 (33) | 1 (33) | 0.76 |
| pH < 7.00 | 0 | 0 | 0 | 0 | 0 | — |
| Sepsis | 2 (3) | 0 (0) | 0 | 0 | 0 | 0.76 |
| Oxygen requirement | 7 (9) | 6 (11) | 2 (9) | 2 (11) | 0 | 0.85 |
| Admission to special-care nursery | 10 (13) | 10 (18) | 2 (5) | 2 (11) | 1 (33) | 0.26 |

[a]Numbers in parentheses indicate percent.

### DISCUSSION

Clinical chorioamnionitis or IAI is now a rare cause of serious maternal morbidity but may result in serious neonatal complications such as sepsis, meningitis, and pneumonia.[6] Additionally, it has been reported that chorioamnionitis may be a cause of intrauterine asphyxia.[13,14] However, Maberry et al.[14] reported no difference in the frequency of acidemia (defined as a Ua pH < 7.20) in the infants of women with labor complicated by chorioamnionitis vs. controls. These authors[14] concluded that asphyxia was a rare event when associated with intrauterine infection, especially in the absence of other signs of fetal jeopardy or ominous FHR patterns.

Nonreassuring FHR patterns are poorly defined and often referred to as fetal distress or fetal jeopardy. While there appears to be general agreement among obstetricians that repetitive severe variable decelerations and repetitive late decelerations necessitate expeditious delivery, there is no similar agreement on the management of other nonreassuring FHR patterns such as decreased variability, fetal tachycardia, or loss of FHR accelerations. Even less is known about the neonatal outcomes associated with these FHR patterns in pregnancies complicated by chorioamnionitis.

In our series, neither the loss of variability nor persistent fetal tachycardia in patients with acute chorioamnionitis who were appropriately treated was associated with an adverse neonatal outcome. Since there were only 8 (4%) fetuses with late decelerations and 7 (3.6%) with severe variable decelerations, we can make no conclusion regarding their significance when associated with acute chorioamnionitis.

In the 8 neonates with late decelerations, only 1 was delivered by cesarean for fetal distress. The other 7 cases had transient late decelerations which resolved with clinical maneuvers to improve uterine blood flow. None of the 8 neonates had a Ua pH < 7.20. The neonatal courses in these infants were universally good. Like others, we considered these patterns ominous if they were persistent, and delivery was accomplished expeditiously in these patients. Finally, we confirmed previous reports that delaying delivery up to 12 h following the diagnosis of chorioamnionitis is not associated with poor neonatal outcome. While there were 3 additional patients who delivered in the 12–18 h interval with uncomplicated neonatal courses, this number is too few to allow us to draw valid conclusions regarding clinical recommendations.

### REFERENCES

1. Gibbs RS, Duff P: Progress in pathogenesis and management of clinical intraamniotic infection. Am J Obstet Gynecol 164:1317–1326, 1991.
2. Gilstrap LC III, Cox SM: Acute chorioamnionitis. Obstet Gynecol Clin North Am 162:373–379, 1989.
3. Newton ER, Prihoda TJ, Gibbs RS: Logistic regression analysis of risk factors for intraamniotic infection. Obstet Gynecol 73:571–575, 1989.
4. Soper DE, Mayhall CG, Dalton HP: Risk factors for intraamniotic infection: A prospective epidemiologic study. Am J Obstet Gynecol 161:562–568, 1989.
5. Looff JD, Hager WD: Management of chorioamnionitis. Surg Gynecol Obstet 158:161–166, 1984.

6. Hauth JC, Gilstrap LC III, Hankins GDV, Connor KD: Term maternal and neonatal complications of acute chorioamnionitis. Obstet Gynecol 66:59–62, 1985.
7. American College of Obstetricians and Gynecologists: Intrapartum fetal heart rate monitoring. Tech Bull No. 132, 1989.
8. Gilstrap LC, Hauth JC, Hankins GD, Beck AW: Second-stage fetal heart rate abnormalities and type of neonatal acidemia. Obstet Gynecol 70:191–195, 1987.
9. Gilstrap LC, Leveno KJ, Burris JS, Williams ML, Little BB: Diagnosis of birth asphyxia on the basis of fetal pH, Apgar score, and newborn cerebral dysfunction. Am J Obstet Gynecol 161:825–830, 1989.
10. Goldaber KG, Gilstrap LC, Leveno KJ, Dax JS: Pathologic fetal acidemia. Obstet Gynecol 78:1103–1107, 1991.
11. Winkler CL, Hauth JC, Tucker M, Owen J, Brumfield CG: Neonatal complications at term as related to the degree of umbilical artery acidemia. Am J Obstet Gynecol 164:637–641, 1991.
12. Kahn H, Sempos C: Statistical Methods in Epidemiology. New York: Oxford University Press, p 193, 1987.
13. Peevey KJ, Chalhub EG: Occult group B strep infection: An occult cause of intrauterine asphyxia. Am J Obstet Gynecol 146:989–990, 1983.
14. Maberry MC, Ramin SM, Gilstrap LC, Leveno KJ, Dax JS: Intrapartum asphyxia in pregnancies complicated by intraamniotic infection. Obstet Gynecol 76:351–354, 1990.
